# A Modified Technique of Temporomandibular Joint Arthroscopic Operative Surgery of the Superior and Inferior Joint Spaces

**DOI:** 10.1007/s12663-019-01291-0

**Published:** 2019-09-30

**Authors:** I. Rosenbrg, A. N. Goss

**Affiliations:** 1 St John of God, Mount Lawley Hospital, Mount Lawley, Australia; 2 Suite 11, Medical Centre, Ellesmere Road, Mount Lawley, WA 6050 Australia; 3 Oral and Maxillofacial Surgery Unit, Faculty of Health Sciences, The University of Adelaide, Adelaide, SA 5005 Australia; 4 The Royal Adelaide Hospital, Adelaide, Australia

**Keywords:** Temporomandibular joint, Arthroscopy, Superior joint space, Inferior joint space, Glenoid fossa, Disc, Condyle, Operative procedure

## Abstract

**Purpose:**

This paper describes in detail the first author's technique of performing arthroscopic surgery in both the superior and inferior joint spaces of the temporomandibular joint.

**Methods:**

The key is careful measurement of sagittal and coronal tomograms to determine the individual size and shape of the joint. The joint is then distracted to allow 3-port video arthroscopy.

**Results:**

The detailed steps in the procedure are described and illustrated.

**Conclusion:**

This modified technique is safe and allows procedures in both joint spaces and surgical access to the fossa, condyle and disc.

## Introduction

Arthroscopic examination of the temporomandibular joint was first described by Ohnishi [1] in Japanese and subsequently in Western literature [2]. Initially, it was described as a diagnostic technique with simple lysis, lavage and medicament insertion.

The technique was then applied to relatively simple operative surgical procedures within the superior joint space [3]. Unless there was a large central disc perforation or absent intra-articular disc, the lower joint space could not be visualised. As at least 50% of the pathology and the predominant site of translational movement is in the inferior joint space, this limited the value of the technique.

- Arthroscopic surgery has now expanded to include multiple procedures: [4].
- Synovectomy in the upper and lower joint compartments.
- Disc repositioning and stabilisation.
- Exploration of the lower joint compartment with lysis and lavage.
- High condylar shave and osteoplasty for chondromalacia and osteoarthritis.

In this paper, we describe, in step sequence, a modified technique of operative surgical approach to the temporomandibular joint, which has been developed over many years by the first author (IR) [5].

## Method

### Preoperative

All TMD patients must have a full evaluation including a medical workup, and non-surgical treatment of splints, exercises, physiotherapy and psychological assessment must have been tried over time and failed. The patient must have demonstrated intra-articular temporomandibular joint pathology as demonstrated by joint tenderness, limitation of opening, difficulty in mastication confirmed by supportive imaging such as CT or MRI scans.

The patient is given a full informed consent of the arthroscopic procedure, including temporary and occasionally permanent facial nerve weakness of the forehead and upper eyelid. The very small risk to the external ear canal and the middle ear also needs to be discussed.

**Fig. 1:**
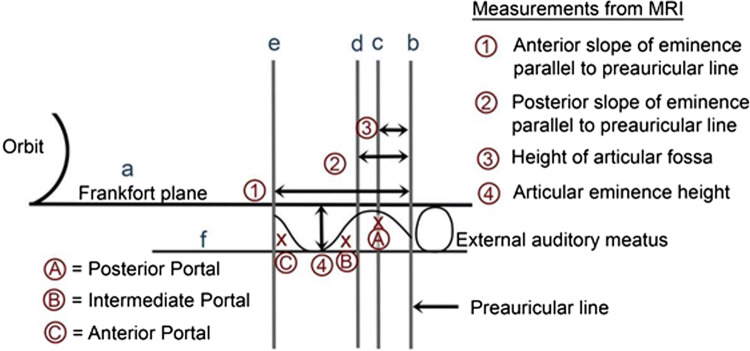
Diagrammatic representation of the skin measurement for TMJ arthroscopic surgery

**Fig. 2:**
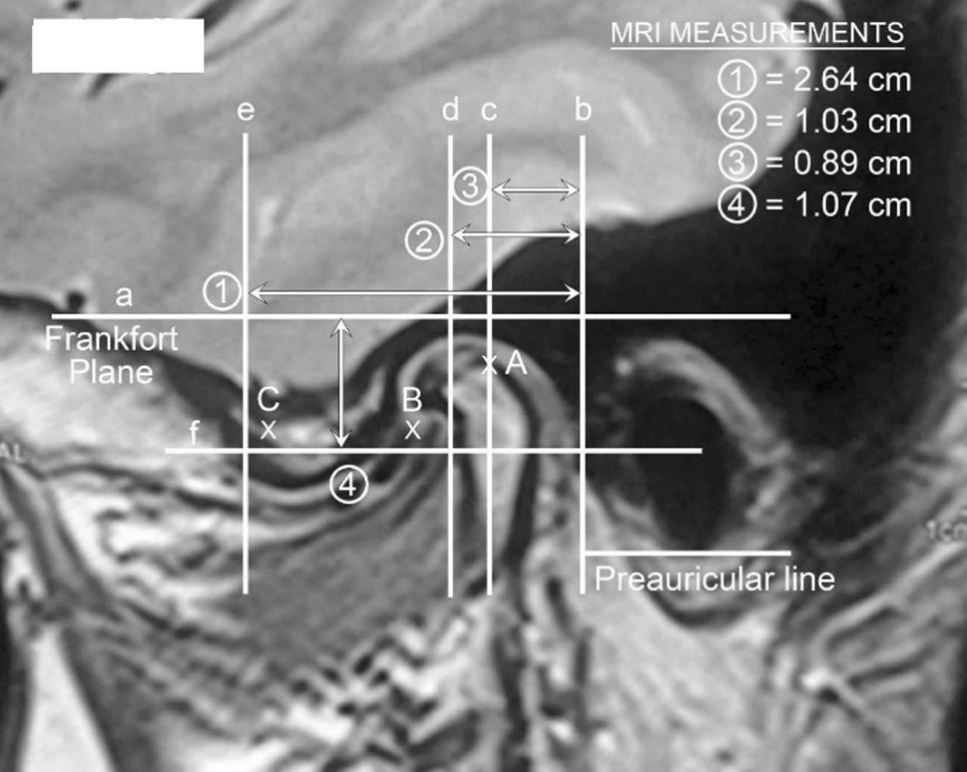
Sagittal MRI showing marking and measurements

Besides examining the MRI and CT scans for diagnosis of the intra-articular pathology, the MRI is carefully examined and measured to determine the precise size of the patients TMJ. These measures are lateral to define the anatomy of the joint and distances both vertically and horizontally to determine the points of insertion of cannulae and instruments (Figs. 1, 2).

### Sagittal Scan

Line (a) is the Frankfort plane which is a horizontal line from the superior margin of the external auditory meatus to the infraorbital rim.

Line (b) is the vertical line immediately anterior to the tragus and in perpendicular to the Frankfort line (preauricular line).

Line (c) is parallel to the preauricular line and passes through the highest point of the glenoid fossa.

Line (d) is parallel to the preauricular line and passes through the posterior limit of the temporal eminence.

Line (e) is parallel to the preauricular line at the anterior limit of the temporal eminence.

Line (f) is a line parallel to the Frankfort plane at the vertical height of the articular eminence.

The following measurements are then recorded.

Measurement 1. Distance from the preauricular line to the anterior eminence line.
  2. Distance from the preauricular line to the posterior eminence line.
  3. Distance from the preauricular line to the highest point of the glenoid fossa.
  4. Distance from the Frankfort horizontal line to the height of the articular eminence.

These measurements are recorded in mm and are computer-derived measurement taken directly off the sagittal MRI scans and available for ready intra-operative reference by the surgeon.

The following points of insertion are defined (Fig. 1).

Point A Along line (c) and into the maximum depth of the glenoid fossa.
Point B At the angle of line (b) and (f).
Point C At the angle of line (d) and (f).

### Coronal Scan

**Fig. 3:**
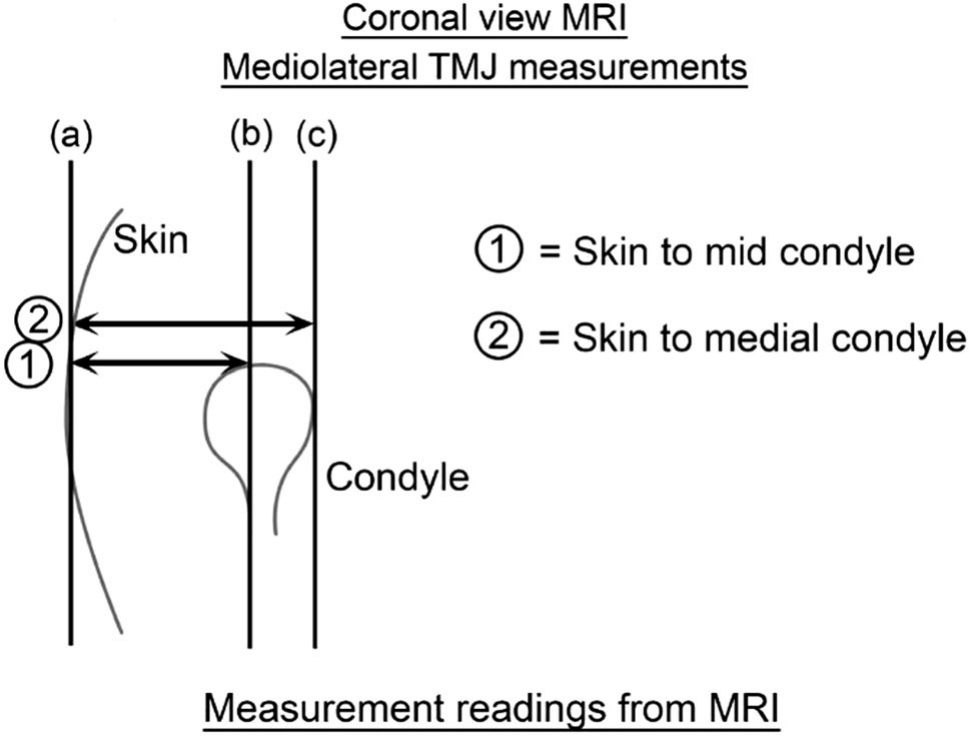
Diagrammatic representation of the coronal TMJ measurements

**Fig. 4:**
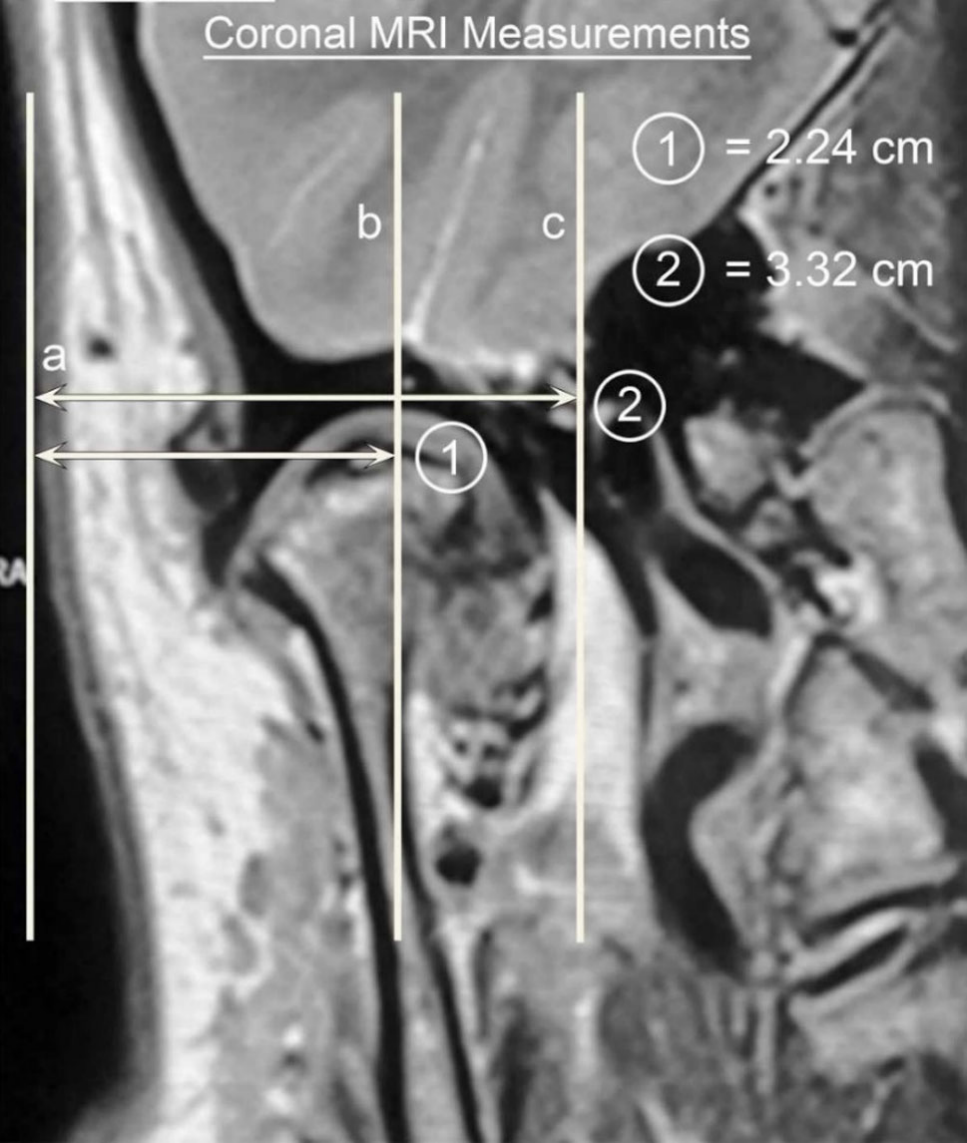
Coronal measures superimposed on the MRI

The coronal scans are next examined and measured for the depth of penetration (Figs. 3, 4).

Vertical lines are drawn at the level of the upper joint space parallel to the skin (Line a), through the mid-condyle (Line b) and to the most medial aspect of the mandibular condyle (Line c). A horizontal line perpendicular to Line (a) is drawn to the mid-condyle and measured in mm, (Line 1) and a second measurement is mm from the skin to medial condyle (Line 2).

The mid-condyle measurements determine the usual working depth and the medial condylar depth the penetration depth beyond which one must not go.

Following the surgical assessment, the patient is seen by the anaesthetist to confirm their fitness and consent for anaesthesia.

### Operative Preparation

At operation, the patient has a nasal intubation either conventionally or by fibre optics, if they have limited jaw opening. Their head is then carefully positioned with the joint to be operated being upwards using a vacuum cushion head support, so the head once positioned cannot move. It is of the utmost importance to the procedure that the head remains completely stable so as not to continually disturb the intra-articular working views. The preauricular region is then shaved minimally, prepared and draped.

**Fig. 5:**
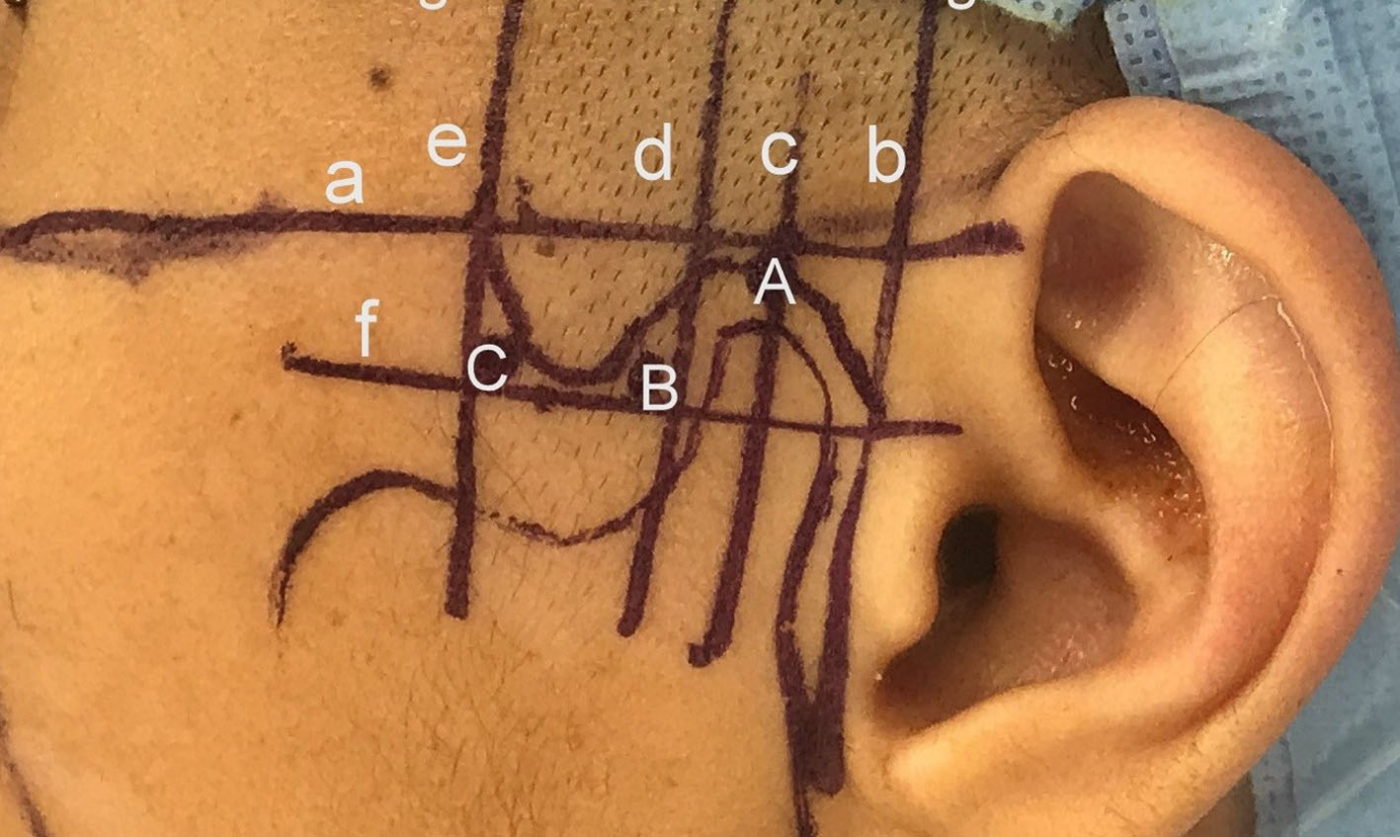
Skin markings

The skin markings, in accordance with Fig. 1, are then made (Fig. 5). A cotton wool plug is then placed in the external auditory meatus. The equipment required is listed in “Instruments and set-up” (Table 1).

**Table 1:** Instruments and set-up

Vacuum head support	Intra-articular instruments^®^
Standard head drape	Blunt and sharp probes
Marker pen	Alligator grasping forceps
Cotton wool ear plugs	Biopsy forceps
Scalpel handle and 11 and 15 scalpel blades	Microscissors
Cannulae 2.7 mm × 40 mm × 2	Irrigation system
Sharp and blunt trochars	Glycine bags 2 l × 2
Fine periosteal elevator	Bupivacaine 0.5% with adrenalin
Small artery forceps (mosquito)	Dexamethasone
Arthroscope 2.3 mm, 30-degree angle (Dyonics)	Prolene suture
Video camera and light source (Dyonics)	Spinal needle, 15 gauge
Monitors ×2	Fogarty balloon
Video printer	Yeates drain tubing
Short cannulae	Silk suture 0000
Suture Jigs	
Electrocautery probes (Bipolar)	
Steinman pins 2 × 25 mm	
Joint distractors	
Electric cordless drill	
Electric handpiece with micro shavers and burrs and foot control^®^	

### Operative

**Fig. 6:**
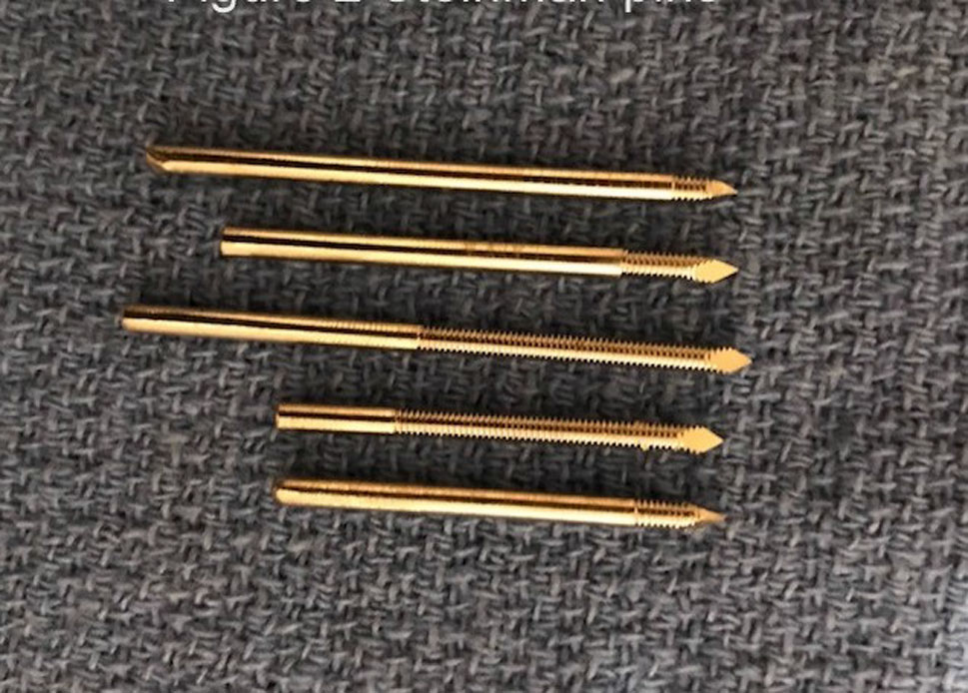
Placement of Steinman pins

The first step surgically is to distract the mandibular condyle inferiorly to maximise the joint spaces. This is performed by placement of two 25-mm-long and 2.4-mm-wide Steinman pins. The first is inserted at right angle to the facial plane into the temporal eminence. A small skin incision is made, and the self-threading Steinman pin inserted to a depth at which the pin feels secure (Fig. 6).

The second Steinman pin is then inserted into the mandible close to the posterior border and approximately 3 cm from the Frankfort plane. The skin is retracted superiorly, a small incision made and the second Steinman pin placed. The superior displacement of the skin prior to pin insertion allows the skin to stretch with the distraction.

**Fig. 7:**
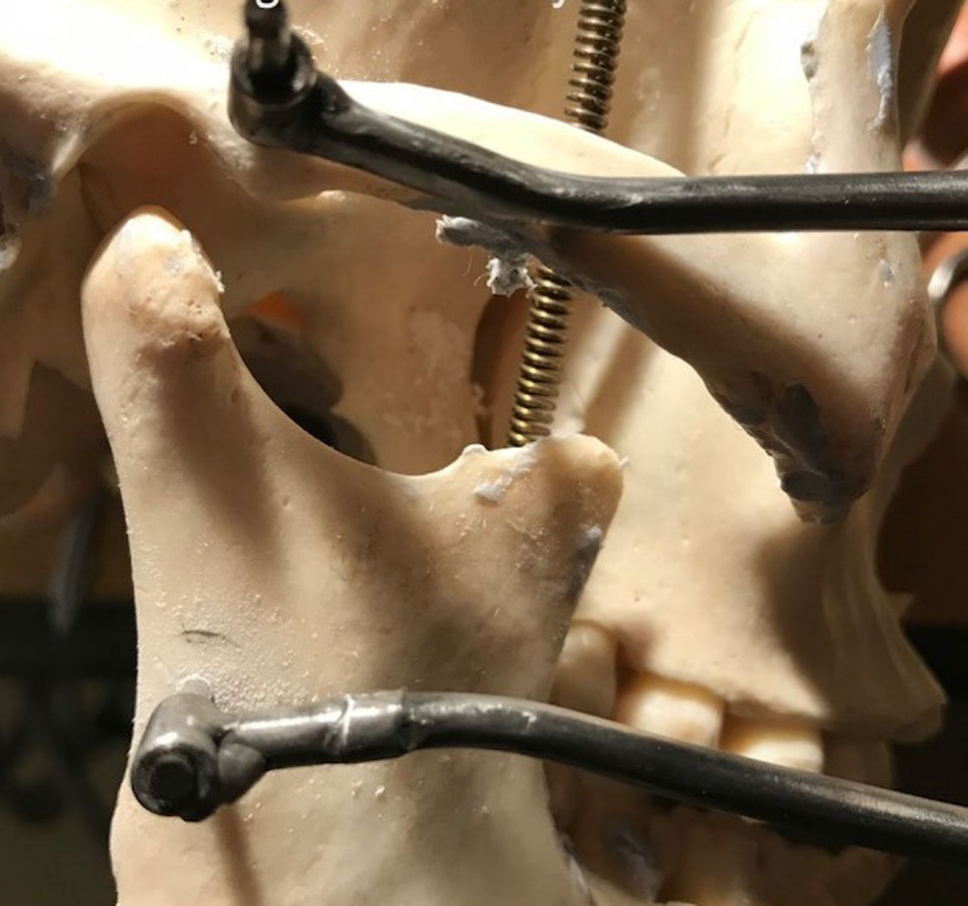
Condylar distraction demonstrated on a skull

**Fig. 8:**
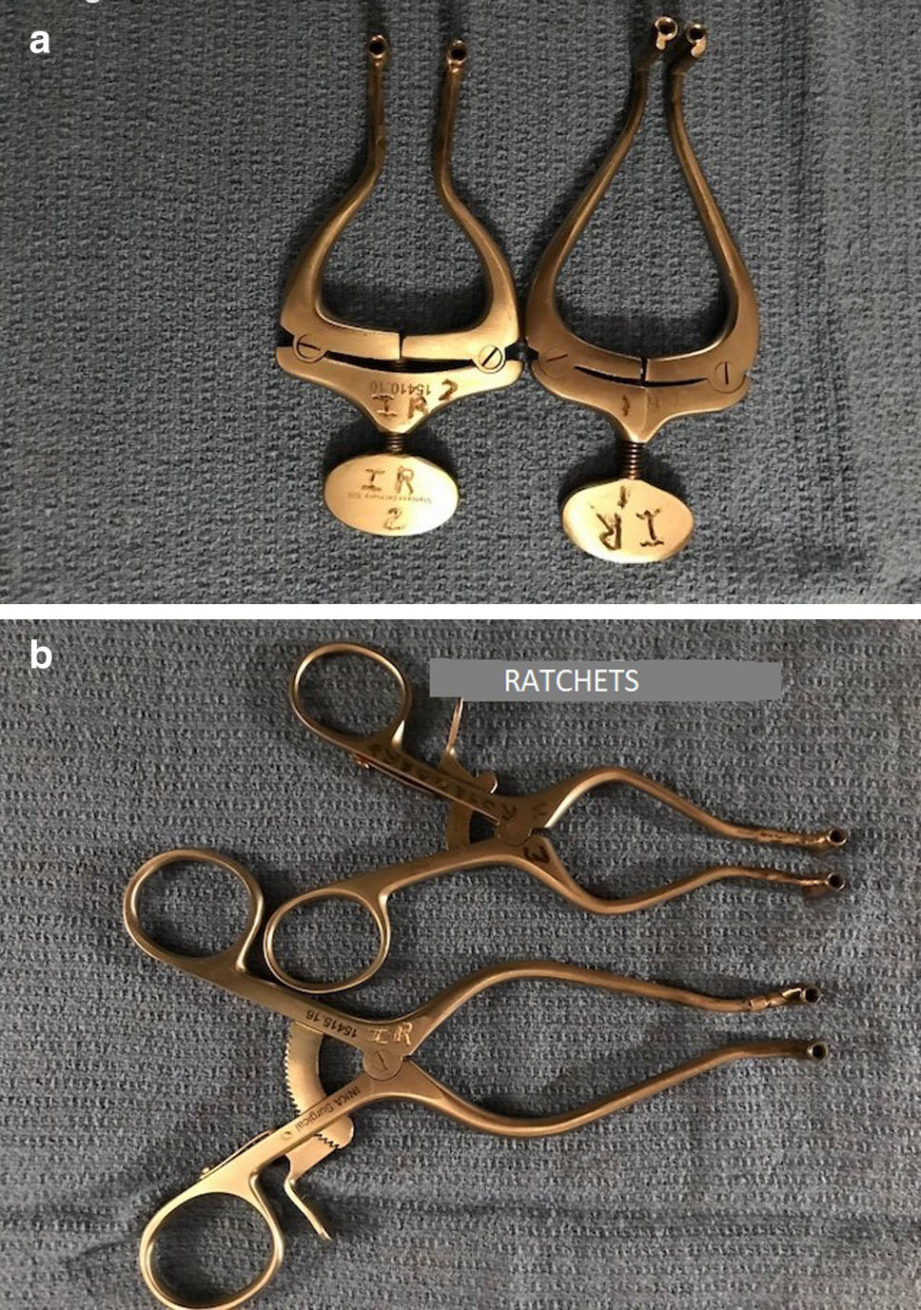
Distraction of the Steinman pins using either a screw distractor or ratchet type distractor

The pins are then distracted using either a hand screw distractor or a ratchet type distractor to the maximum possible with great care (Figs. 7, 8a, b).

An intra-articular injection of 2 ml of bupivacaine is then made into the posterior compartment of the superior joint space. The needle is inserted directly into the upper joint space on line (c) (Fig. 1) just below the rim of the glenoid fossa as determined by palpation. The aim of the injection is to distend the superior joint space further and for anaesthesia and vasoconstriction of the bilaminar zone.

**Fig. 9:**
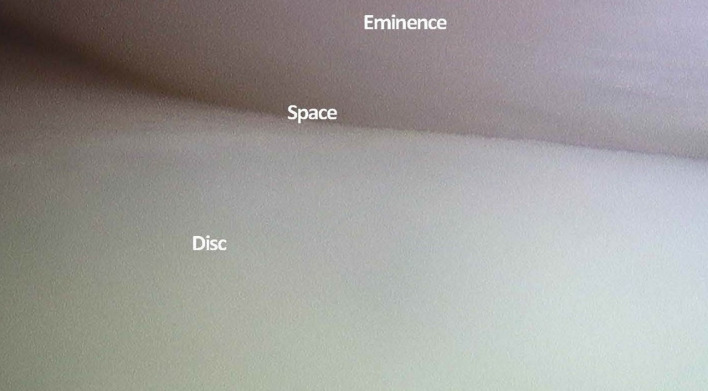
Arthroscopic view of the superior joint space

The first sharp cannula and trochar are inserted at point A. An initial small 3-mm skin incision is made, and the trochar inserted at an upwards, forward and medial trajectory. A 19-gauge needle is inserted alongside the cannula for irrigation. The sharp trochar is withdrawn, and the 2.7 mm 30° rod lens video arthroscope placed into the cannula. A detailed examination of the superior joint space is now performed (Fig. 9).

This examination gives an initial impression of the extent of the intra-articular pathology of the superior joint space involving the eminence, glenoid fossa and the intra-articular disc.

**Fig. 10:**
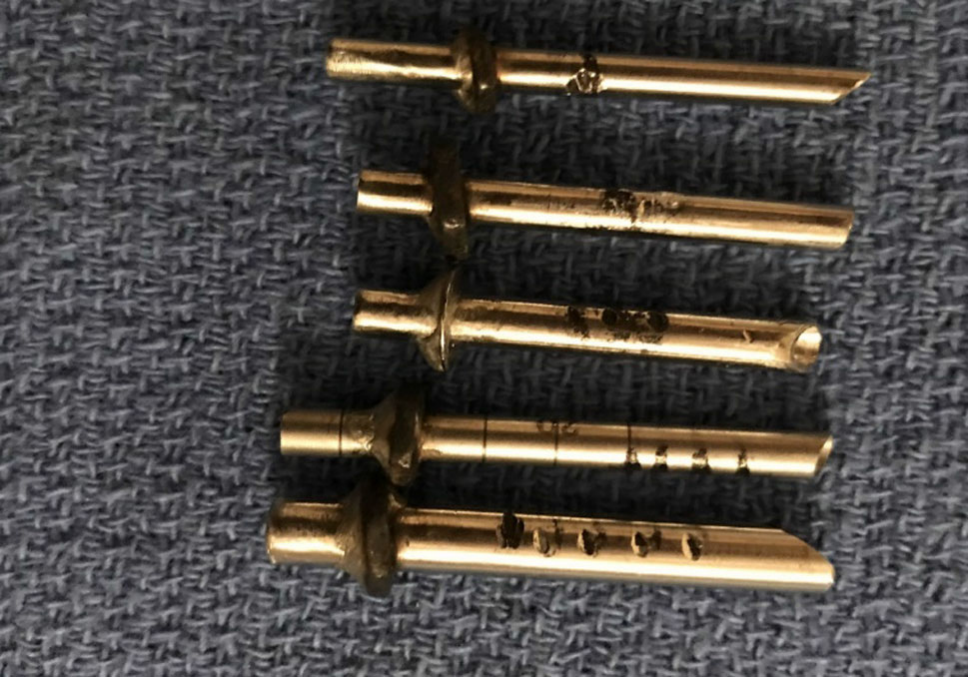
Short cannulae to be inserted at point B

**Fig. 11:**
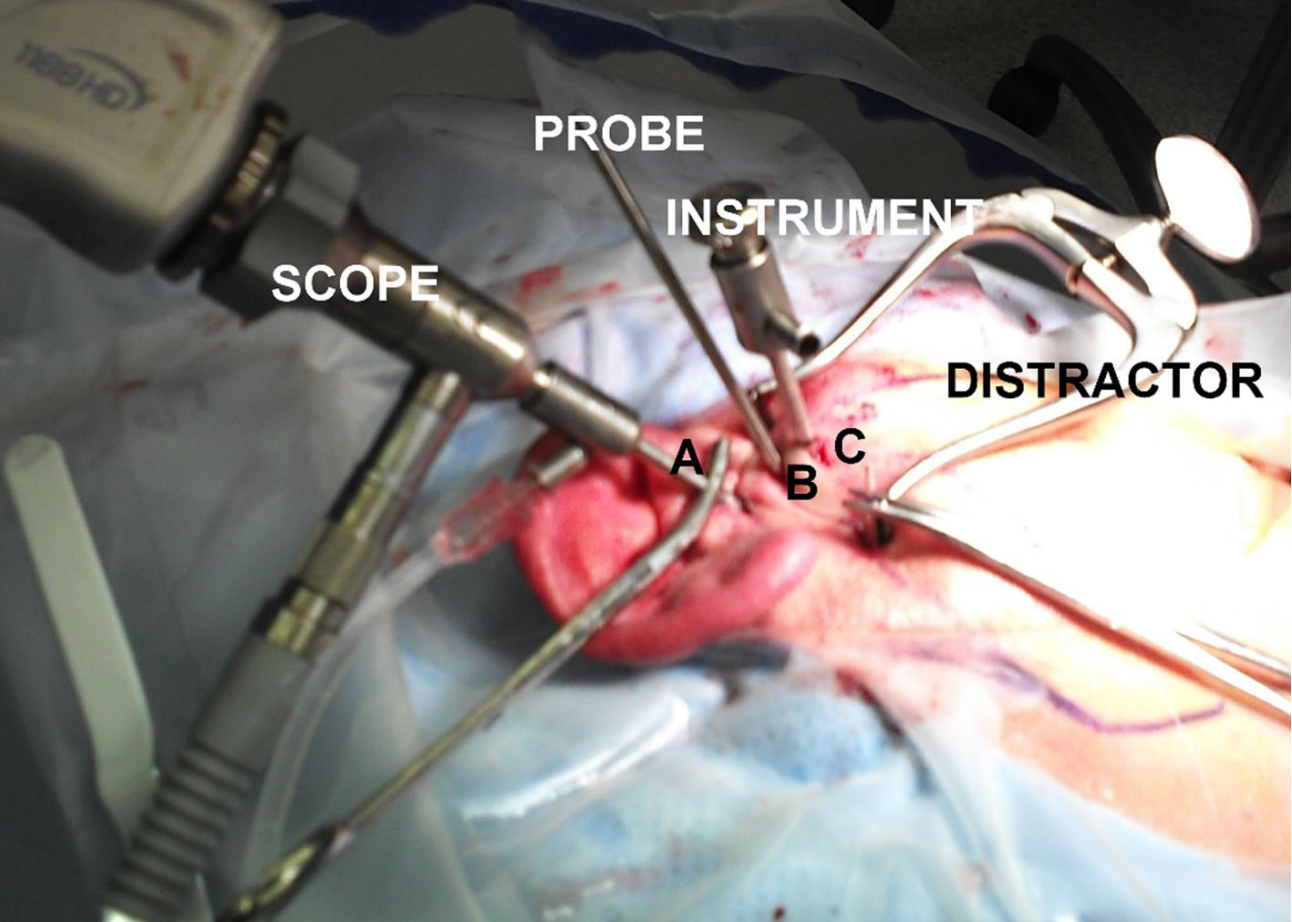
Three portal arthroscopic set-ups

The second entry point is then made at point B, which is at the posterior slope of the articular eminence. A small 3-mm incision is made through the skin, and a short cannula and trochar are inserted (Figs. 10, 11).

Portal B allows for disc manipulation with a probe and facilitates unobstructed insertion of the third cannula at point C. The third entry point is then made at point C. A small 3-mm incision through the skin is made at the anterior slope of the articular eminence, and a 2.7-mm cannula and sharp trochar inserted. This allows instrumentation for several operative procedures to be performed. At all times the sharp trochar is replaced with a blunt trochar to avoid trauma to the intra-articular structures.

Intra-operative surgical procedures are given as follows.

**Fig. 12:**
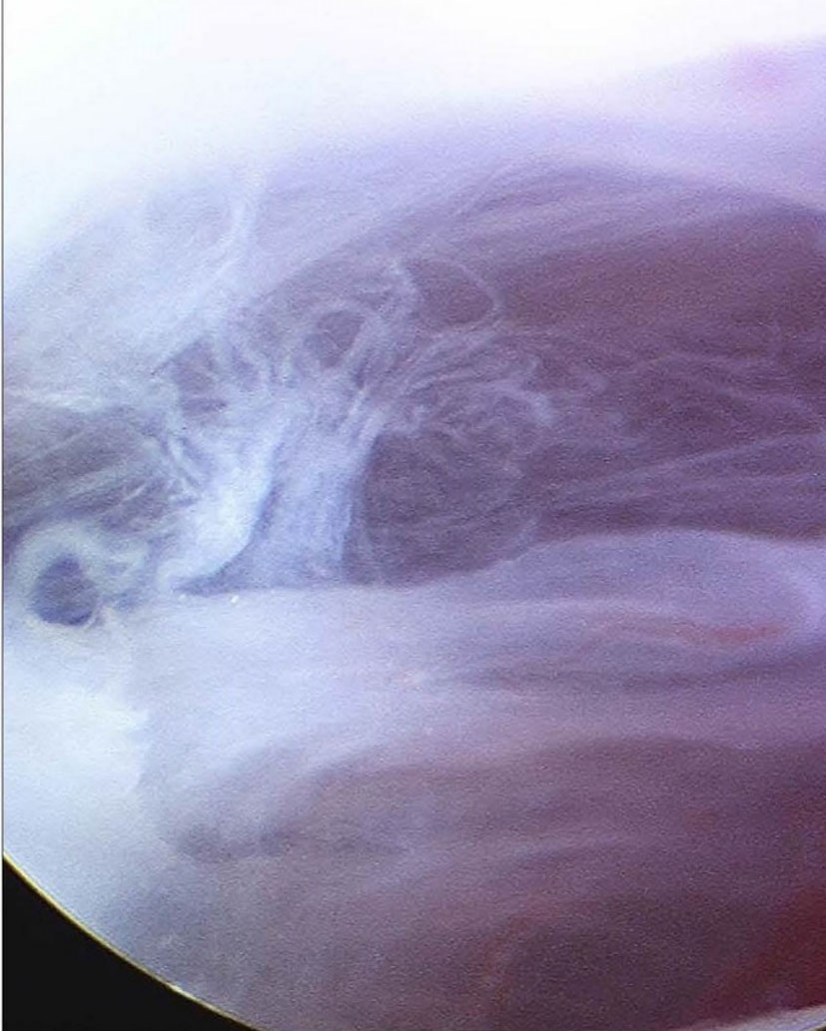
Fine adhesions
  The superior joint space is washed out continuously with glycine. The fine adhesions are disrupted and inflammatory products washed out. The joint is then manipulated to determine whether a normal range of movement can be achieved.
  Dexamethasone 2 ml and bupivacaine .5% 2 ml are then placed and the arthroscopic procedure completed. This procedure is equivalent to a simple arthrocentesis [6].

**Fig. 13:**
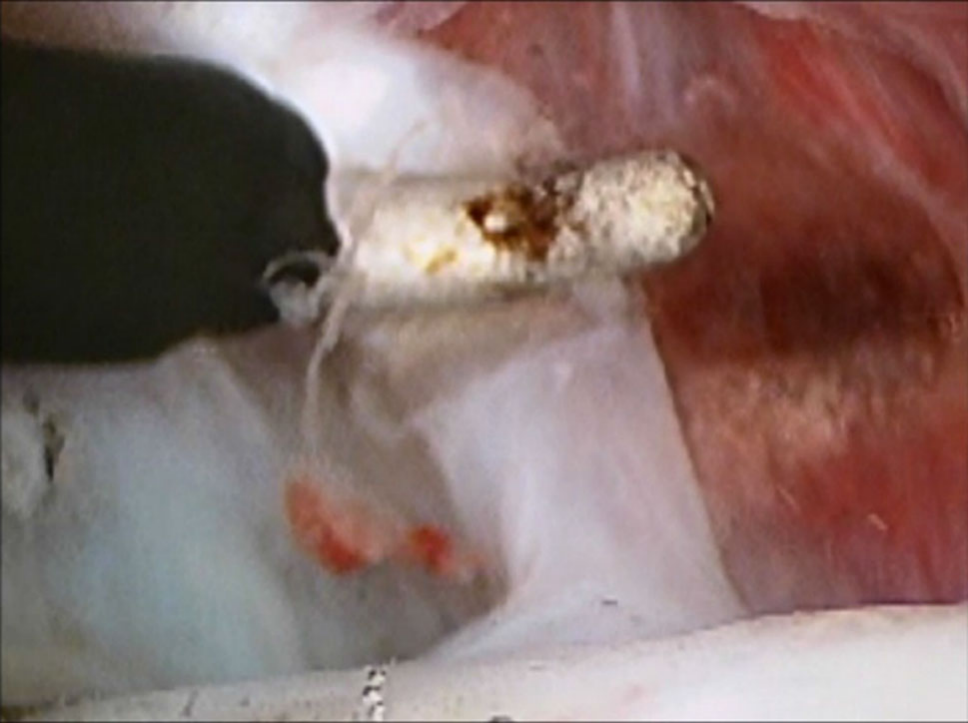
Cauterisation of thick fibrous bands. *Not included*

**Fig. 14:**
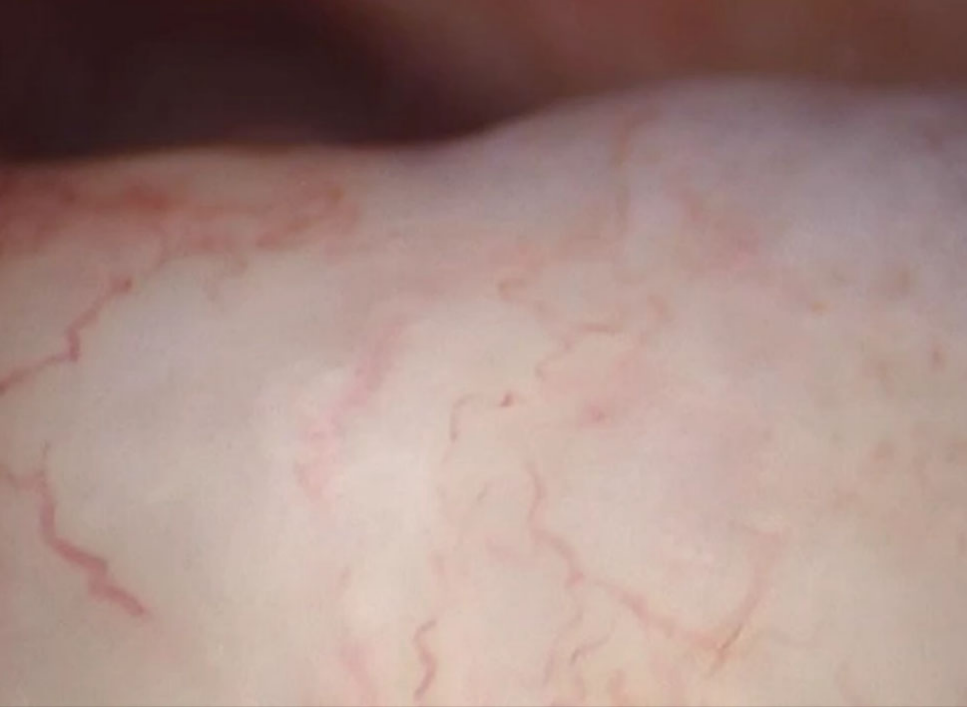
Synovitis with hyperaemia

**Fig. 15:**
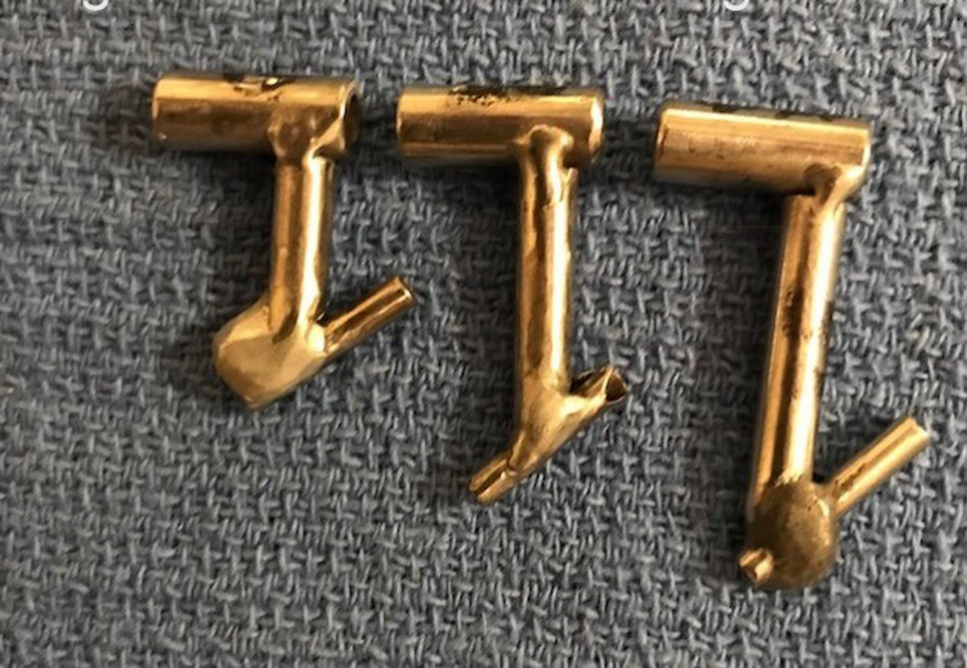
Disc suturing jigs

**Fig. 16:**
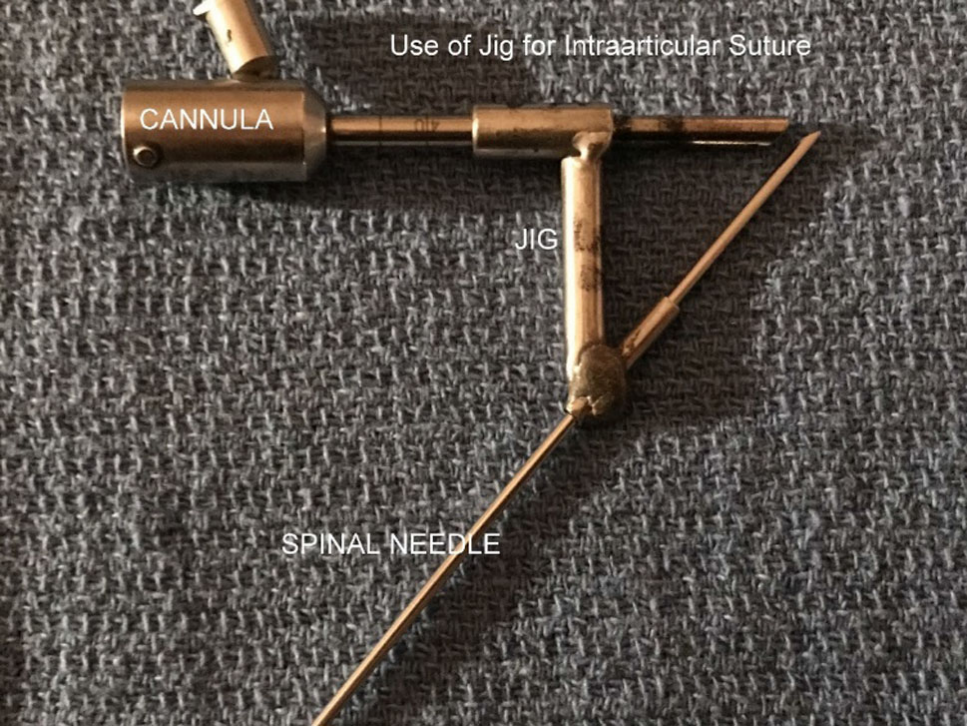
Jig for the intra-articular suture

**Fig. 17:**
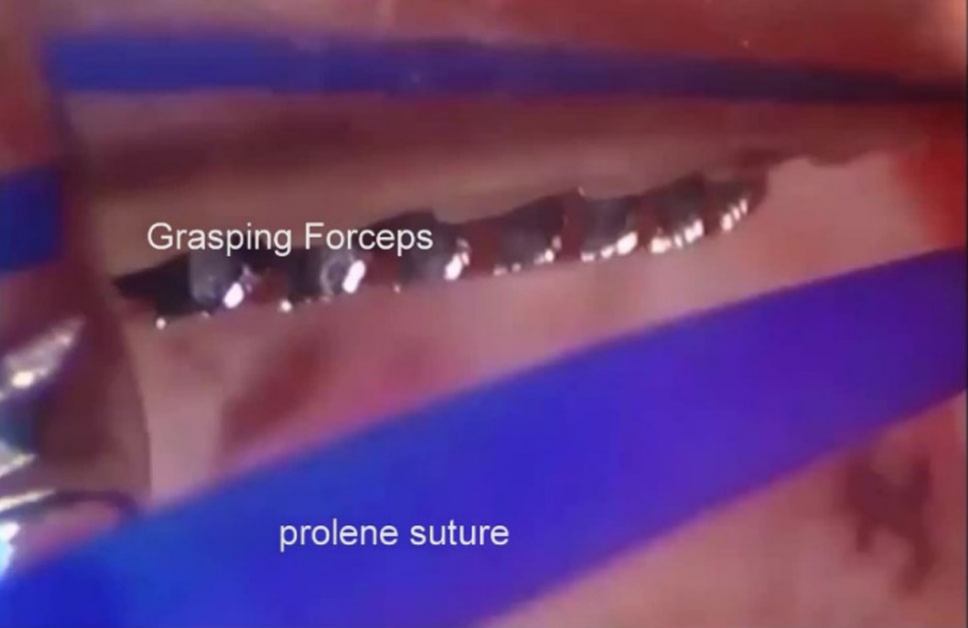
The repositioning suture is grasped in the superior joint space with an alligator forceps
  An awl with a single 0 prolene suture is passed subcutaneously through the cartilaginous component of the external auditory meatus. The other end of the suture is then also passed subcutaneously through the cartilage of the external auditory meatus approximately 1 cm lateral to the first limb of the suture using an awl. Both limbs of the suture are held together and pulled posteriorly to determine whether they exert movement on the disc (Fig. 18). The limbs are now temporarily clipped to the drapes.

- Superior joint space lysis and lavage.
  This is the simplest procedure and is based on the finding that the initial examination has demonstrated fine fibrous adhesions across the superior joint space (Fig. 12).

(b) Superior joint synovectomy.
  If examination of the superior joint spaces shows thick intra-articular adhesions and synovial inflammation, then firstly the thick fibrous bands are cauterised to free them (Fig. 13) and then partial synovectomy performed by electrocautery of the involved areas of synovitis (Fig. 14).

(c) Posterior disc repositioning.
  If the disc is anteriorly displaced and non-reducing, then the disc should be repositioned as best as possible and stabilised. An extensive cauterisation of the posterior attachment of the disc is carried out. An extensive cauterisation of the posterior attachment of the disc is carried out. Similarly, the posterior aspect of the roof of the glenoid fossa is cauterised so that once the disc is repositioned this allows reattachment of the bilaminar zone to the roof of the fossa once the endaural repositioning suture is placed.
  The endaural repositioning suture is placed as follows: A switching stick is used to facilitate the withdrawal and reinsertion of the cannula through portal A. The jig is now used to place the endaural plication suture. Three sizes of jig are available dependent on the depth of the arthroscope in the joint (Fig. 15). A gauge spinal needle and prolene suture is placed initially through the skin, near the external auditory meatus, low down posterior to the disc and passed up through the disc or the bilaminar zone, as far anteriorly as possible into the upper joint space using the jig (Fig. 16). The suture is then grasped in the superior joint space with grasping alligator forceps placed through portal C (Fig. 17).

**Fig. 18:**
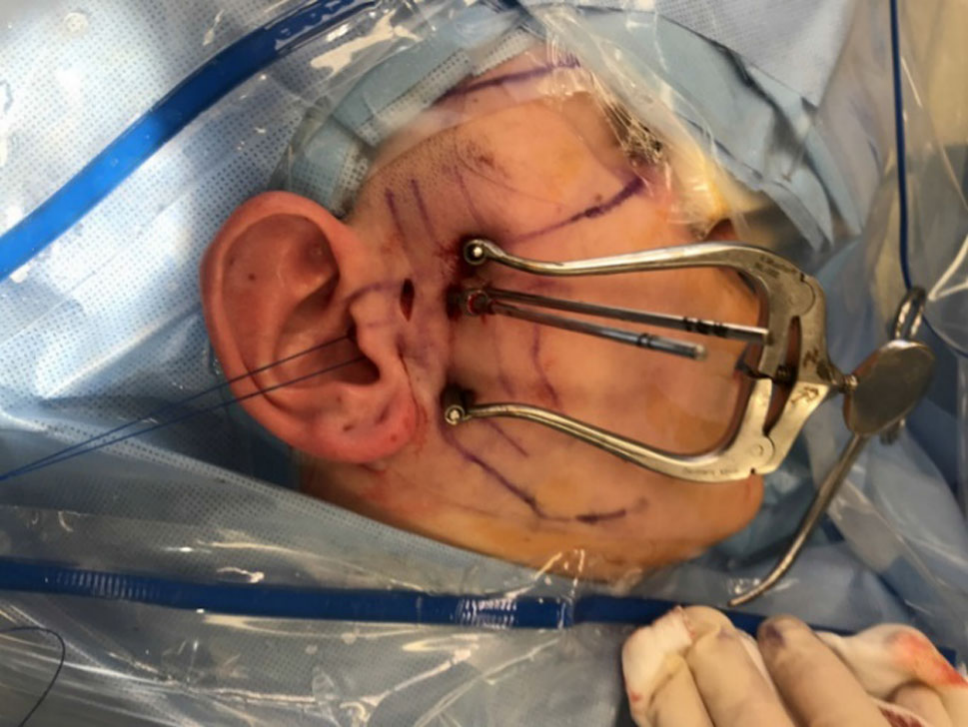
Placement of the endaural repositioning suture

**Fig. 19:**
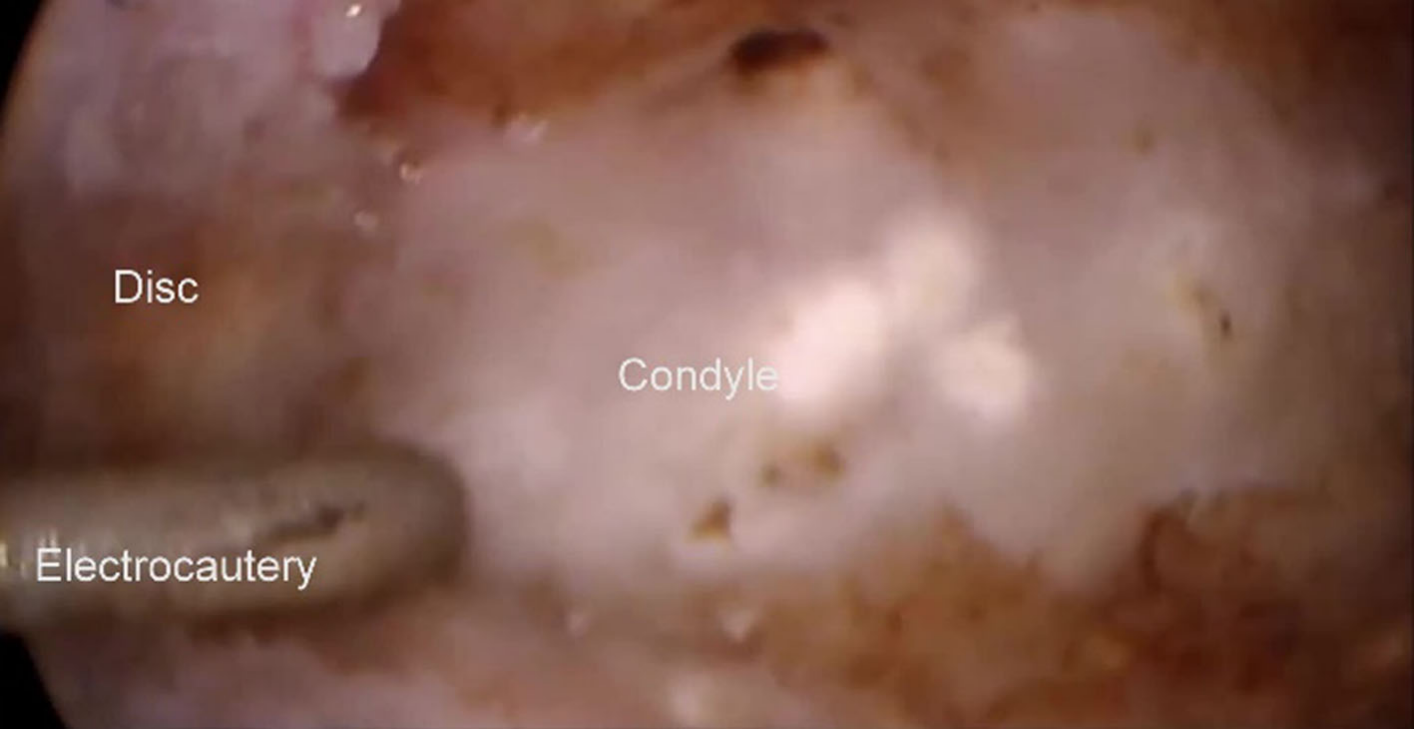
Examination of the inferior joint space
  Depending on the findings, various procedures can then be carried out including lysis and lavage, cauterisation of fibrous bands and an electrosurgical shaver to carry out a high condylar shave (Fig. 20) and electrosurgical burrs (Fig. 21) to achieve limited osteoplasty.

(d) Inferior joint procedures.
  If the disc cannot be reduced or if the preoperative examination has shown significant inferior joint space pathology the inferior joint space is accessed.
  This involves an anterior release incision in the disc extending laterally and posteriorly to incise the disc, entering the lower joint compartment, without disturbing the attachment of the disc to the capsular ligament. This allows access into the inferior joint space by the arthroscope to allow firstly detailed examination of the inferior joint space with the video arthroscope (Fig. 19).

**Fig. 20:**
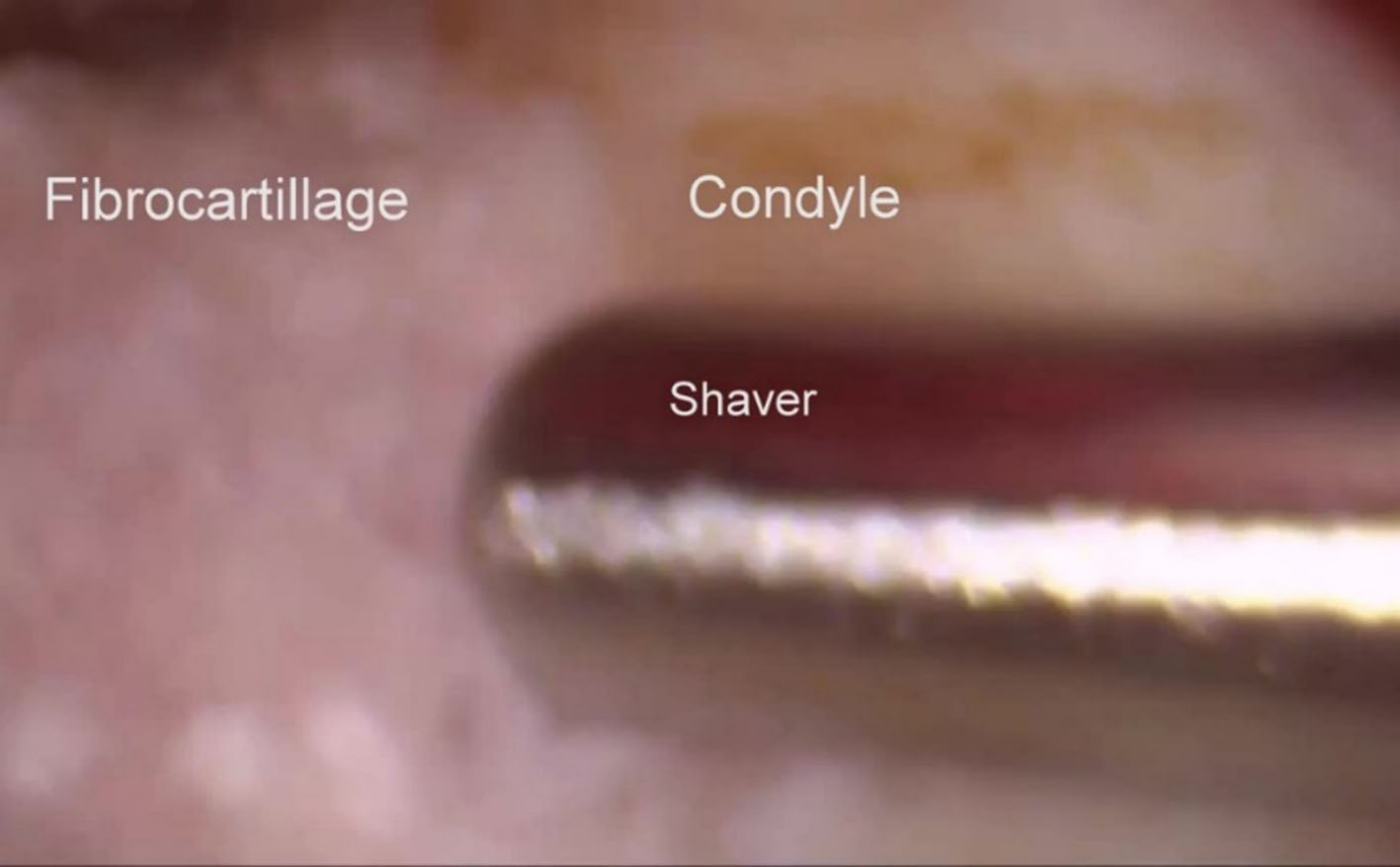
High condylar shave using the electric shaver

**Fig. 21:**
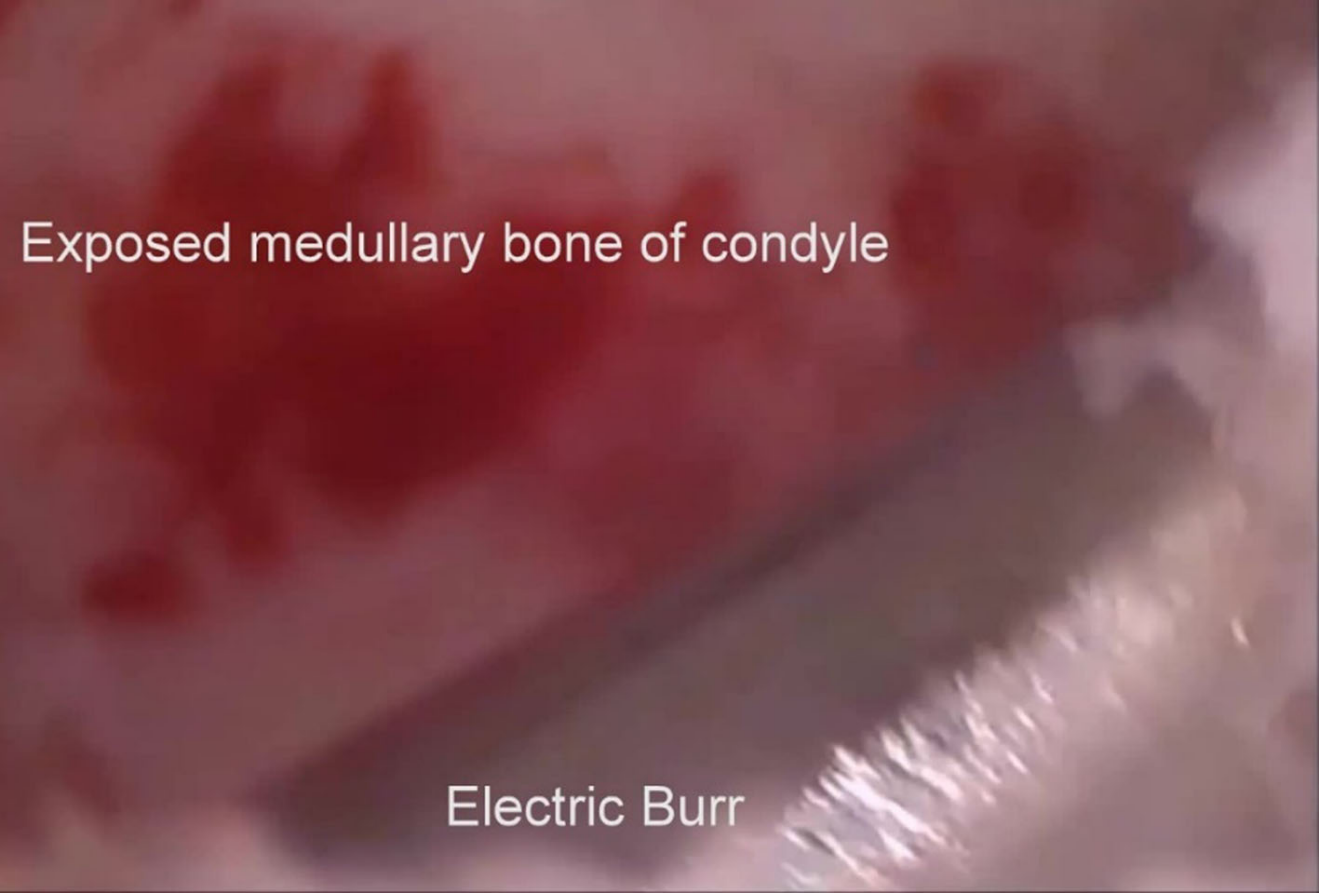
High condylar shave using an electrosurgical burr
  This surgery is performed under direct vision of the arthroscope placed via portal A. The disc is laterally retracted via portal B to secure the disc, while an incision into the disc is made and operative instruments are placed and manipulated via portal C.
  On completion of the procedures in both upper and lower joint compartments, fragments are irrigated out via portals B and C.

(e) Disc reposition and suture.
  This is performed as the end step of the operative procedures.
  The disc is repositioned posteriorly as best as possible with a probe via portal B. The
  Prolene application mattress suture is now tied within the ear canal with tension. A small segment of a Yates drain is placed over the endaural section of the suture to prevent the suture from cutting through the ear cartilage in the initial healing period. The purpose of this suture is to retain the repositioned disc in position until adequate fibrous adhesion takes place between the cauterised superior surface of the posterior attachment and the roof and posterior surface of the glenoid fossa.
  At this point, the distractor is removed so that the condyle returns to its normal position and the mandible is moved to determine that there is movement between the condyle and disc and between the disc and articular eminence and fossa. It may not be possible to retract the disc to its original anatomical position due to the fact that the disc itself may be deformed and the posterior attachment fibrosed. What is important however is to determine that there is movement in the joint as described above.

(f) Final procedures.
  The distractor is replaced and intra-articular steroid dexamethasone 2 ml and bupivacaine 0.2% 2 ml are inserted into the upper and lower joint space via portal C. The joint spaces are finally checked via portal A with the video arthroscope and then the arthroscope removed. The distractor is removed, followed by the Steinman pins. Fine sutures (0000 silk) are placed as needed through the portals and pin placement incisions.

(g) Bilateral procedures.
  The second joint is operated as needed.

### Postoperative

The anaesthetic is completed and then nasal tube removed. Ice packs are placed with mild pressure. Normal postoperative observations are performed for a few hours as required.

Fit patients operated in the morning are discharged 4–6 h postoperative. Medically unfit patients, extensive procedures or if there is inadequate postoperative care at home, are admitted overnight.

#### Follow-up

Patients are reviewed weekly with removal of skin sutures at 1 week. The endaural suture is removed at 2 weeks. This is performed under a preauricular local anaesthetic just anterior to the external auditory meatus. At 3 weeks, physiotherapy and replacement of the occlusal splint is commenced.

Patients are seen monthly until fully recovered.

### Complications

Complications are best minimised by careful attention to surgical technique. The joint should not be penetrated deeper than the coronal measurement from skin to medial condyle. Also, insertion of the sharp trochar at point A is directed medially and anteriorly and never posteriorly as this can result in middle ear damage.

The most common complication is neuropraxia of the frontal branch of the facial nerve. This usually resolves in up to 3 months. The nerve however may be more seriously injured by the sharp trochar at point A or through the placement of the superior Steinman pin. Although potentially permanent, this had not occurred in the author’s personal experience of the technique.

All patients must be preoperatively be given valid informed consent of the risk of these complications.

## Discussion

Arthroscopic surgery of the temporomandibular joint is a complex procedure which requires careful attention to surgical detail. For this reason, we have carefully described the technique in particular defining the anatomy of the joint and portals of entry into the small anatomically complex joint.

There have been a number of excellent papers and texts on arthroscopic surgery, and serious students of the techniques are recommended to study them all [1–12].

The initial studies of Ohnishi [1] and Goss and Bosanquet [2] were designed to describe the technique of diagnostic arthroscopic surgery of the superior joint space. They described the basic technique and procedure. The papers of Holmlund [7] and Tarro [8] provided different skin markings and entry points to our technique. The main reason for this is they used standardised measurements for all cases, whereas in our technique we individualised the measurements based on the MRI image of each patient. They also only entered the superior joint space. Similarly, Monje [9] and McCain [10] use slightly different trochar and cannula placements.

The release of the disc by an anterior incision follows that described by McCain [4, 10], and the placement of the endaural repositioning suture follows that presented by Michael Koslin [11, 12].

Arthroscopic surgery is minimally invasive and avoids significant skin scars and greater risk of damage to the facial nerve. We commend it to surgeons interested in TMJ surgery.

## Abbreviations

TMD: Temporomandibular disorders
TMJ: Temporomandibular joint
CT: Computerised tomography
MRI: Magnetic resonance imaging
